# Prospective prediction of PTSD diagnosis in a nationally representative sample using machine learning

**DOI:** 10.1186/s12888-020-02933-1

**Published:** 2020-11-10

**Authors:** Michelle A. Worthington, Amar Mandavia, Randall Richardson-Vejlgaard

**Affiliations:** 1 Department of Psychology, Yale University, New Haven, USA; 2 Department of Counseling and Clinical Psychology, Teachers College, Columbia University, New York, USA

**Keywords:** Post-traumatic stress disorder, Machine learning, Nationally representative

## Abstract

**Background:**

Recent research has identified a number of pre-traumatic, peri-traumatic and post-traumatic psychological and ecological factors that put an individual at increased risk for developing PTSD following a life-threatening event. While these factors have been found to be associated with PTSD in univariate analyses, the complex interactions of these risk factors and how they contribute to individual trajectories of the illness are not yet well understood. In this study, we examine the impact of prior trauma, psychopathology, sociodemographic characteristics, community and environmental information, on PTSD onset in a nationally representative sample of adults in the United States, using machine learning methods to establish the relative contributions of each variable.

**Methods:**

Individual risk factors identified in Waves 1 of the National Epidemiologic Survey on Alcohol and Related Conditions (NESARC) were combined with community-level data for the years concurrent to the NESARC Wave 1 (*n* = 43,093) and 2 (*n* = 34,653) surveys. Machine learning feature selection and classification analyses were used at the national level to create models using individual- and community-level variables that would best predict the new onset of PTSD at Wave 2.

**Results:**

Our classification algorithms yielded 89.7 to 95.6% accuracy for predicting new onset of PTSD at Wave 2. A prior diagnosis of DSM-IV-TR Borderline Personality Disorder, Major Depressive Disorder or Anxiety Disorder conferred the greatest relative influence in new diagnosis of PTSD. Distal risk factors such as prior psychiatric diagnosis accounted for significantly greater relative risk than proximal factors (such as adverse event exposure).

**Conclusions:**

Our findings show that a machine learning classification approach can successfully integrate large numbers of known risk factors for PTSD into stronger models that account for high-dimensional interactions and collinearity between variables. We discuss the implications of these findings as pertaining to the targeted mobilization emergency mental health resources. These findings also inform the creation of a more comprehensive risk assessment profile to the likelihood of developing PTSD following an extremely adverse event.

## Background

The ability to predict the onset of post-traumatic stress disorder (PTSD) remains an important goal for clinicians and researchers. In the United States, it is estimated that 51.2 to 60.7% of population are exposed to at least one extremely adverse event in their lives and approximately 1 to 12.3% of those individuals will go on to develop PTSD [1, 2]. Experiencing an extremely adverse event (sometimes referred to as a potentially traumatic event, or a traumatic event) event is a necessary criterion for a diagnosis of PTSD [3] but is insufficient to explain its eventual onset [4]. Recent research has examined and identified a number of pre-traumatic, peritraumatic and post-traumatic psychological and ecological factors that put an individual at increased risk for developing PTSD following a life threatening event [5–8]. While these factors have been found to be associated with PTSD in univariate analyses, the complex interactions of these risk factors and how they contribute to individual trajectories of the illness are not yet well understood [9, 10].

In adults, psychological and ecological risk factors known to be associated with PTSD include a history of extremely adverse life events, socio-demographic variables, social support factors, history of psychopathology, and environmental factors. A number of studies have shown that childhood physical abuse, neglect, and sexual abuse are associated with the development of PTSD in adulthood [11–14]. These early life events may contribute to an increased level of emotional and physiological arousal which has been associated with the development of PTSD in response to an event later in life [14]. Pre-existing sociodemographic factors and later life events for the development of PTSD include female gender [15, 16], non-white race/ethnicity [17–19], lower IQ and educational attainment [8], unemployment [20, 21], history of incarceration [22, 23], intimate partner physical abuse [11] and history of suicide [24].

Regarding history of psychopathology, research has shown a relationship between depression and PTSD among those who have experienced interpersonal violence, injury resulting in emergency room visits, natural disasters, and childhood maltreatment [25–27]. Other studies have shown an increased risk of developing PTSD for individuals diagnosed with either a mood disorder or a personality disorder [5, 28, 29]. Substance abuse has also been shown to be a risk factor for PTSD as well as commonly co-occurring with PTSD following a traumatic event [30]. Interpersonally, lack of social support in close family members, friends, and neighbors prior to a traumatic event has been linked to the development of PTSD in numerous populations following a traumatic event [28, 31, 32].

There is increasing evidence that neighborhood- and community-level factors play a role in risk for PTSD. These factors may interact with individual-level factors to comprise an individual’s risk profile. Living in close proximity to a natural disaster or terrorist attack may increase an individual’s risk for developing PTSD following the event [33, 34]. It has also been shown that exposure to community violence in childhood is a risk factor for developing PTSD later in life youth living in both urban and suburban neighborhoods [35]. Further, childhood trauma may interact with the neighborhood crime rates to predict PTSD later in life [36]. From an economic standpoint, a higher level of income inequality in an individual’s resident state has been shown to be a risk factor for PTSD [37]. In addition, socio-demographic factors may contribute more to risk of PTSD in high-income countries as compared to low-income post-conflict countries where citizens have been chronically exposed to public violence (e.g. during apartheid regime in South Africa) [38].

With the wealth of data available to study individual and epidemiological risk for developing PTSD, statistical methods such as machine learning are ideal for managing large amounts of data and detecting complex interactions between risk factors. As outlined in Dwyer et al. [39], machine learning approaches can be incredibly useful in the fields of clinical psychology and psychiatry when employed appropriately and are especially well-suited to PTSD given the heterogeneous risk profiles, types of events and individual trajectories that have been identified [40]. The analytical process typically involves a feature selection process, which statistically selects a number of variables from a large data set that have the greatest influence on the outcome of interest, the creation of a regression or classification algorithm, and the validation of the algorithm in an independent dataset or in an unused subset of the original data [39]. Multiple studies have used machine learning approaches to understand the development of PTSD using smartphone data [41], neuroimaging biomarkers [42], and data from longitudinal emergency room studies in adults [43, 44] and children [45].

In the present study, we used an exploratory machine learning approach to examine the relationships between individual variables and community-level variables in predicting the onset of PTSD. We incorporated data from two waves of a longitudinal nationally-representative epidemiological survey—which includes demographic information, individual trauma exposure, social support, and psychopathology—with publicly-available data about crime rates, educational attainment, employment rates, and regional economies. We sought to determine how well classification algorithms using these variables could detect new onset of PTSD at the second wave of the survey and which factors had the strongest influence.

## Methods

### Data

Data were taken from the National Epidemiological Survey on Alcohol and Related Conditions (NESARC) Wave 1 (*n* = 43,093; 2001–2002) and Wave 2 (*n* = 34,653; 2004–2005). Data at both time points was collected using a face-to-face, computer-assisted personal interviews conducted in participant’s residence. We combined the two waves based on unique individual identifiers which yielded a final sample of 34,653 individuals, with data present for both waves. For more information on recruitment, refer to Hasin and Grant [46]. Community-level data on crime, education, household income, GDP, and employment in each regional census division (i.e. New England, Mid-Atlantic, East North Central, West North Central, South Atlantic, East South Central, West South Central, Mountain and Pacific) were collected from the websites of various government agencies.

### Individual measures

The NESARC assesses for psychiatric conditions based on the DSM-IV criteria using the Alcohol Use Disorder and Associated Disability Interview Schedule–DSM–IV Version (AUDADIS– IV) [47]. This tool is administered as a diagnostic semi-structured interview by graduate level researchers and assesses a number of substance use, mood, anxiety, and personality disorders. Diagnosis from the DSM-IV were further classified based on time course of the onset of the illness, in relation to the PTSD diagnosis at Wave 2, using data from both waves. Distal factors included lifetime diagnosis and diagnosis prior to Wave 1, using data from Wave 1. Proximate factors included diagnoses that had an onset between the two waves, using data from Wave 2. Specifically, change in status from wave 1 to wave 2 was used as a predictor in the classification model for predicting new onset of PTSD diagnoses at Wave 2. Therefore, proximal diagnoses were classified based on onset: 1) since Wave 1 (i.e. in the 3 years between Waves 1 and 2); 2) since Wave 1, but before the last year (i.e. onset between years 1 and 2 after Wave 1); and 3) in the past year (i.e. 3 years after Wave 1). For the purpose of this study demographic, substance use disorder, traumatic events, suicidality, social support, help-seeking behaviors & DSM-IV-TR axis I and II diagnosis (i.e. mood, anxiety, and personality disorders) variables were included. These variables were chosen based on the factors identified in the existing literature on risk for PTSD. Diagnoses of PTSD in the AUDADIS-IV is consistent with DSM-IV-TR diagnostic principles which specify (Criterion A) having been exposed to (i.e., experienced, witnessed, or confronted with) a serious life threatening event (e.g., “Were you EVER in a serious or life-threatening accident? Were you EVER sexually assaulted, molested or raped or did you EVER experience unwanted sexual activity?”). Respondents were asked to identify the event that was the MOST stressful, and then to report on their emotional reactions to this event (fear, helplessness etc.), any psychological sequelae, the latency of onset of these symptoms, and their duration. The diagnosis of PTSD in the current study was determined by a positive response to criterion A and a positive response on at least two of the subsequent criteria (B, C or D) as defined in the DSM-IV-TR (i.e., re-experiencing the event (Criterion B), avoidance of related stimuli (Criterion C), persistent emotional arousal (Criterion D). The validity of AUDADIS diagnosis of PTSD is comparable to the Clinically Administered PTSD Scale (CAPS-DX), which is widely considered the ‘gold standard’ for PTSD diagnosis in the field (Positive Predictive Value = 0.75, Negative Predictive Value = 0.97) [48].

### Contextual variables

For both time points (2001–2002 and 2004–2005) we collected data on contextual variables in the wider community that could have an effect on the rate of PTSD diagnosis in that community. Specifically, we included change in contextual variables between 2001 through 2005. Data on crime rates (number of crimes per 100,000 persons), prevalence of violent crimes (e.g., murder, rape, robbery, assault), property crime (e.g., burglary, larceny, and motor vehicle theft) were collected from the FBI uniform crime reports for each census region. The unemployment rate, per capita income, gross domestic product, number employed, and active labor force were extracted from the US Department of Commerce Bureau of Economic Analysis. Data regarding the total population and the percent of individuals above 25 years of age that have graduated high school was collected from the Department of Education. In all, 134 discrete variables were included in the analysis.

### Feature selection

All statistical analyses were performed in R [49]. The first step in the analytic process, called “feature selection” was conducted using a “Gradient Boosting Machine” (GBM) to select the predictor variables from the total number of variables that would be included in the final model. This approach uses regression principles including least squared error to perform consecutive iterations of predictive models (learners) to find the optimal fitting model [50]. The GBM evaluates the derived models by conducting a k-fold cross-validation, where *k* was defined as the number of groups the training dataset further split into. These subgroups within the training dataset are then evaluated using the derived training model and used to refine the choice of variables to be included in the final model. We fit the GBM to conduct a 10-fold cross-validation, based on recommendations by Natekin and Knoll [50]. The output of GBM ranks variables by order of relative influence in the model on the outcome variable, (new PTSD diagnosis at Wave 2). Variables that had a relative influence of 0.5 or greater were included in the classification models that were subsequently built. This boosting method is advantageous because it prevents model over-fitting, by selecting random start points from which to begin the iterative selection process. As such, this method is able to more systematically identify the best fitting model.

### Classification algorithms

The second step in the analytic process involves training classification models (described below) in the training data using the selected features using. To ensure that the model derived using the GBM were not an artifact of the method, but instead was a meaningful predictors of new PTSD onset, we used three methods to determine which algorithm best classified the testing data using the model derived from the GBM: classification trees, penalized logistic regression, and Bayesian Additive Regression Trees (BART) machine, which used 10-fold cross-validation in the training process. Classification trees are built using recursive partitioning to systematically identify a subset of predictors based on how significantly each predictor contributes to the model. These classification trees are often advantageous for interpretability; however, other methods typically perform better in predictive accuracy [51]. A penalized logistic regression improves predictive accuracy by applying a penalty to non-zero coefficients, ultimately minimizing the sum of squared error. This method is useful to prevent overfitting when working with data that features high dimensionality or collinearity [51, 52]. The BART machine approach uses multiple trees within the same model, otherwise known as ensemble-of-trees. This approach is able to detect interactions, nonlinearities, and missing data with enhanced accuracy. Because BART is a tree-based approach as opposed to a regression-based approach, it is the best equipped of the three methods to handle data with high dimensionality (containing a vast number of predictor variables), nonlinear outcome and predictor variables, and interactions in predictors, without increasing the error coefficient [53]. Overall, these methods are better equipped to handle large datasets with a large amount of missing data and allow for the use of both regression and classification analysis in the same analysis, which reduces the likelihood of Type I errors.

To test the models described above, the data were split into training sets and testing sets. A subset of 10,000 cases was randomly selected as the training set. The remaining 24,653 cases comprised the testing data. The models were built using the training data and were tested in the testing data for prediction accuracy. To assess the classification ability of these algorithms, we computed accuracy, sensitivity, and specificity scores for each method to predict PTSD at Wave 2 [54].

### Data analysis

Community-level data was mapped onto individual-level data by the respective census division that was reported at Wave 1 and Wave 2. This allowed for examination of regional differences in individual- and community-level predictors of PTSD across time; with the outcome variable being defined as a new onset of PTSD between Wave 1 and Wave 2. As the main outcome of interest is new diagnoses of PTSD, to prevent conflating past diagnosis of PTSD with new onset cases, we classified individuals with a diagnosis of PTSD prior to Wave 1 as those without a new onset PTSD diagnosis, while still including them in the analysis. Thus, individuals with a past history of PTSD who had a reoccurrence of PTSD between Wave 1 and 2, were not classified as new cases, but instead treated as preexisting PTSD cases and included in the analysis. To prevent over-fitting of the data, we first ran a Gradient Boosting Machine, which is a conservative feature selection algorithm to identify variables that have a relative influence of 0.5 or greater. After selecting the most salient variables, we cross-validated our results by implementing three different classification methods (i.e. tree classification, penalized regression, & BART). Due to the limited number of variables extracted, we report all factors derived in the GBM model. Although these analyses are in many ways redundant, the purpose of using these three approaches was to ensure that the results we received were consistent across two or more methods, and to identify potential method-specific bias. Consistency in results across methods as assessed by the accuracy, sensitivity, and specificity of classification indicates stability in the models that were built. These ML methods identify salient predictors, however, they do not indicate the direction of their effects; as such, we conducted post-hoc chi square tests of the variables that were identified to determine whether associations were positive or negative.

## Results

A total of 2785 individuals met criteria for new-onset PTSD diagnosis at wave 2. The three classification methods predicting new-onset PTSD at wave 2 performed with 92.03 to 95.09% accuracy, with sensitivity ranging from 92 to 97.7% and specificity ranging from 0 to 67.7%. For specifics on each model, refer to Table 1. Overall, the BART algorithm provided the best accuracy (95.09%), sensitivity (97.7%), and specificity (67.7%).

**Table 1:** Performance of machine learning algorithms

Algorithm	Accuracy	Sensitivity	Specificity
BART^a^	95.09%	97.7%	67.7%
Logit	92.3%	93.3%	55.8%
Tree	92.03%	92.0%	0.0%

^a^Bayesian Additive Regression Trees

The relative influence of predictor variables from the GBM are reported as percentages of the overall variance accounted for by that variable. The variables were grouped into domains including: DSM-IV diagnosis, community factors, demographic characteristics, social support, and exposure to extremely adverse events. A report of descriptive statistics and relative influence reports are presented in Table 2.

**Table 2:** Distribution and relative influence of significant variables

	Risk Factors	No PTSD Diagnosis (***n*** = 31,868)		PTSD Diagnosis (***n*** = 2785)		Chi-square Value	Relative Influence ^a^
		n	%	n	%		
DSM-IV Diagnosis	Major depressive episode, lifetime	6287	19.73	1653	59.35	2274.6*	10.38
	Major depressive episode, since last interview	2513	7.89	1060	38.06	2518.5*	17.29
	Major depressive episode, since last interview, before past year	1728	5.42	766	27.50	1866.5*	0.19
	Manic episode, since last interview	519	1.63	343	12.32	1201.6*	0.19
	*Mood disorder total*						*28.05*
	Generalized anxiety disorder, lifetime	1897	5.95	833	29.91	2022.2*	1.97
	Generalized anxiety disorder, since last interview	966	3.03	584	20.97	1924.6*	1.40
	Generalized anxiety disorder, past year	844	2.65	519	18.64	1728.2*	0.08
	Panic disorder with Agoraphobia, since last interview	156	0.49	191	6.86	1041.5*	0.09
	Social Phobia, since last interview	698	2.19	371	13.32	1057.7*	0.07
	Specific phobia, since last interview	2237	7.02	739	26.54	1240*	1.01
	Specific phobia, past year	2069	6.49	689	24.74	1161.6*	0.38
	*Anxiety disorder total*						*5.0*
	Schizotypal Personality Disorder, Lifetime	984	3.09	550	19.75	1676.5*	0.41
	Borderline personality disorder, lifetime	1409	4.42	822	29.52	2673.3*	47.92
	*Personality disorder total*						*48.33*
Trauma Exposure	Ever stalked by anyone	1523	4.78	579	20.79	1138.2*	0.11
	Other than terrorist attack, ever have someone close to you die unexpectedly	12,413	38.95	1766	63.41	618.83*	0.07
	Ever sexually assaulted, molested, raped, or experienced unwanted sex	2387	7.49	941	33.79	2017.3*	17.61
	Physically attacked/beaten/injured before age 18 by parent/caretaker	913	2.86	407	14.61	952.95*	0.09
	Seriously neglected before age 18 by parent/caretaker	818	2.57	377	13.54	914.18*	0.08
	Ever physically attacked/beaten/injured by spouse or romantic partner	1864	5.85	709	25.46	1414.6*	0.66
	*Traumatic exposure total*						*18.62*

* *p* < 0.0001, ** *p* < 0.001

^a^ Relative influence is reported as percent influence

The domains that predicted the onset of PTSD between Wave 1 and 2 were DSM-IV diagnosis (81.38%), and adverse event exposure (18.62%). Within the DSM-IV diagnosis domain, personality disorders accounted for 48.33% of the relative influence, mood disorders accounted for 28.95% of the relative influence, and anxiety disorders accounted for 5% of the relative influence. We found that distal diagnostic risk factors such as lifetime diagnosis of Borderline Personality Disorder (BPD, 47.92%), Major Depressive Disorder (MDD, 10.38%), and Generalized Anxiety Disorder (GAD, 1.97%) account for the majority of the relative risk influence. These distal diagnostic risk factors, including those with a relative influence less than 0.5%, account for 60.87% of the total relative influence on the development of PTSD between Wave 1 and Wave 2 (See Table 2).

More proximal diagnostic risk factors, i.e., those with an onset between the two waves, included a diagnosis of Major Depressive Disorder in the past year at Wave 2 (17.29%), and Generalized Anxiety Disorder diagnosis (1.40%) and Specific Phobia (1.01%) between Wave 1 and 2. These proximal factors, in addition to those with a relative influence less than 0.5%, account for 20.7% of the relative influence upon development of PTSD between Wave 1 and Wave 2.

Among the extremely adverse events, having ever experienced unwanted sex (17.61%) and physical abuse by a romantic partner (0.66%) were the only events with a relative influence greater than 0.5%. As a whole, exposure to extremely adverse events accounted for a total of 18.62% of the relative influence on the diagnosis of PTSD.

In summary, the variables with the highest relative influence were a lifetime diagnosis of Borderline Personality Disorder (BPD) in the DSM-IV diagnosis domain (47.9%) and experience of unwanted sex, e.g. assault, molestation, and rape (17.6%) in the extremely adverse event domain. There was no significant impact of community, demographic, and social support variables, nor any Wave 1 variables upon prediction of new onset of PTSD between Waves 1 and 2.

## Discussion

In this study, we performed an exploratory analysis using machine learning techniques to identify factors that could prospectively predict the onset of Post-Traumatic Stress Disorder in a large nationally representative sample. Our results showed that an existing diagnosis of Borderline Personality Disorder was the strongest predictor of a new PTSD diagnosis in a 3-year follow-up period, with a depressive episode during that period as the second strongest predictor. These two disorders therefore contributed almost 60% of the relative influence on a new diagnosis of PTSD in individuals who did not report such a diagnosis 3 years earlier. This finding is not entirely surprising, given the number of studies that have found high levels of comorbidity among PSTD, Borderline Personality Disorder and Major Depressive Disorder [55–58]. What was surprising was the comparatively small effects attributed to community-based predictors. Our analysis found that person-level, proximate variables had a stronger association to the development of PTSD. More distal, community-based factors had very weak associations. This may be due in part to peculiarities of our data. We used US Census, Department of Commerce, and Department of Education data to capture community-level features such as income level, crime rate, unemployment level, and educational attainment. The Census divisions are created to be comparable in terms of gender, age, race, educational attainment, income across 9 broad regions, as such local or state differences could be minimized as a result of this aggregation. It is therefore possible that if each locality or state were compared directly to each other, greater variability (and stronger associations with the outcome) might be seen.

Whereas lifetime exposure to extremely adverse events is fairly common across the world, with estimates ranging from low of 54% of the general population in Japan to a high of 73% of the population in South Africa, the lifetime prevalence of PTSD is estimated to occur in about 2–3% of the population [38]. It is therefore essential to understand what factors may be associated with the likelihood of developing the disorder. Our analysis revealed that a prior diagnosis of Borderline Personality Disorder or Major Depressive Disorder confers a major proportion of the risk of receiving a subsequent diagnosis of PTSD, suggesting that existing psychopathology may be part of a causal pathway to PTSD, as described in the diathesis-stress model proposed by Mckeever and Huff [59]. According to this model, a prior diagnosis of BPD or MDD may alter the threshold for which an extremely adverse event is experienced as traumatizing, thereby contributing to the subsequent emergence of PTSD. Despite a high prevalence of exposure to extremely adverse events in the 3-year follow-up period in this sample, these exposures had considerably lower relative influence on the diagnosis of PTSD than those associated with other forms of psychopathology.

The differences in the relative influence of pre-existing psychopathology versus extremely adverse events in the emergence of PTSD (60% vs 19%) suggests an attenuated effect of adverse events in the aetiology of PTSD. This notion has been documented in epidemiological studies comparing the rates of “traumatic” events to the rates of PTSD, however it has not previously been demonstrated in a large-scale prospective study. Our analysis suggests that the relevance of the stressful event in the aetiology of PTSD appears to be of considerably less importance than the existing psychopathology of the individual. It is well established that up to two thirds of people who experience a life threatening or extremely stressful event do not go on to develop PTSD symptoms [3]. Our finding suggest further, that a significant majority of the subset of individuals who develop PTSD following an adverse experience will have had a history of Borderline Personality disorder, and/or Major Depressive Disorder. The proportion of people without a history of psychiatric diagnosis is small, suggesting that it is somewhat rare to encounter an individual who meets DSM-V criteria for PTSD who has not had a prior psychiatric condition. The difference in relative influence between distal (60.87%) and proximal factors (20.7%) in the aetiology of PTSD suggest that experiencing trauma is not the major contributor to the development of PTSD. Because of the lack of prospective studies in the literature on trauma and its effects, the conflation of cause and effect in the phenomenology of PTSD has been perpetuated. The current study is the only large scale prospective epidemiological study to date examining factors that contribute the emergence of new diagnoses of PTSD.

It is also possible that the co-occurrence of BPD, MDD and PTSD indicate a commonly shared aetiology. These disorders are all characterized by emotional lability and experiences of emotional distress, causing some nosologists to propose combining them into a diagnostic category of “distress disorders” that is distinct from other forms of mental illness such as fear disorders (e.g., obsessive compulsive disorder, panic) or thought disorders (e.g., schizophrenia spectrum disorders [58]. The theoretical rationale for Kotov et al.’s [58] distinction was based on an approach to taxonomy in which features of disorders were classified using factor analytic methods. It was found that MDD, PTSD, and BPD characteristics clustered together phenomenologically and statistically, indicating considerable overlap in their clinical presentation.

Despite a high prevalence of exposure to extremely adverse events in the 3 year follow-up period, these exposures had considerably lower relative influence on the diagnosis of PTSD than those associated with other forms of psychopathology. Exposure to extremely adverse events contributed about 17% of the relative influence on the emergence of PTSD. With 89.7–95.6% prediction accuracy, our models performed very well when incorporating numerous environmental, individual, demographic, and psychological factors. Post-hoc chi-squared tests of independence revealed significant differences for the most influential variables in each major variable category between those with PTSD at Wave 2 and those who did not have PTSD at Wave 2 (Table 2). Within the trauma exposure variable category, the following variables were the strongest predictors: “ever sexually assaulted, molested, raped, or experienced unwanted sex”; “other than a terrorist attack, ever have someone close to you die unexpectedly”; and “ever stalked by anyone.”, the following variables were strongest: “ever attempted suicide”; total personal income; and origin or descent. Within the social support variable category, the following variables were strongest: “If I were sick, I know I would find someone to help with my daily chores”; “Before age 18, felt that someone in my life believed in me”; “Felt there was someone in the family who wanted me to be a success”; and “Before age 17, felt that my family was a source of strength and support.” In the community factors category, the following variables were strongest: annual unemployment rate, total population, and rape rate.

In a separate analysis of data from the Wave 2 cohort of this study, Pagura et al. [60] found that lifetime prevalence of BPD among those with a diagnosis of PTSD was 24.2%. The number of individuals reported in Pagura et al. (2010) was 2463, however in our analysis the number of individuals with a lifetime diagnosis of PTSD was found to be 3621. Pagura et al. [60] used a more restrictive definition of “traumatic event” specifying that each PTSD symptom must be related to the event in question. The approach used in our analysis is consistent with DSM-IV criteria, in which the symptoms occur following a traumatic event, but are not specifically tied causally to the event in the individual’s mind. Although Pagura’s is more conservative, ours is more consistent with current diagnostic standards.

Exposure to extremely adverse events contributed about 17% of the relative influence on the emergence of PTSD. This finding draws into focus the role that extremely adverse events play in the aetiology of PTSD, and consequently the use of the term “traumatic event”. It is often unclear in the research and clinical literature whether the term “trauma” refers to a quality of an event that occurs (i.e. destructive, intense, menacing), or the experience of the individual who endures it (i.e., fearful, psychologically jarred). Uncertainty about the objective or subjective determinants of this term may inadvertently contribute to assumptions about the psychological effects of intense, destructive or threatening events, which are typically referred to as “traumas” [29, 61]. If an event is described as “a trauma” or “traumatic” then the logical reaction of any person enduring it is to “be traumatized”. Current research on subjective reactions to destructive or extremely adverse events does not however bear out this association [62]. For example, a large systematic review by Neria, Nandi and Galea [63] showed that 60–70% of individuals who are directly exposed to life-threatening disasters do not report symptoms of PTSD, and 90 to 95% of individuals in the general population who experience disasters do not report symptoms of PTSD.

Our ML method of analysis that prospectively predicts the emergence of PTSD has significant implications for the screening, diagnosis and treatment of PTSD and related disorders, particularly in situations of national emergency. This approach to understanding a multiply determined phenomenon such as PTSD offers a systematic way to best identify at risk populations, and to mobilize emergency mental health resources in a timely way.

Currently the best supported treatment for PTSD includes exposure therapy, empirical support exists for the efficacy of behavioral activation in the treatment of depressive disorders, and Dialectical Behavior Therapy has gained significant support in the treatment of Borderline Personality Disorder. Clinicians would be well advised to consider the shared influence of BPD and MDD in the emergence of PTSD, and consequently consider altering treatment guidelines to respond to the specific symptom presentation of patients, rather than using general diagnostic categorizations when making treatment recommendations.

To our knowledge, this is the first large-scale, longitudinal study with prospective design examining the emergence of new PTSD diagnosis in a general population sample. For this reason it offers unique insights into the aetiology of PTSD that other research has struggled to clarify. It was nonetheless impossible with the available data to determine the temporal relevance of exposure to extreme stressors on the emergence of PTSD, as the incidents captured at Wave 2 of the NESARC survey did not specify a time frame for these events. As such the stressors reported in this study could have been recent or distal, and may not have been directly associated with the diagnosis of PTSD as recorded in the AUDADIS-V.

While the results from this study are an encouraging step toward understanding risk factors for the development of PTSD, there are several important limitations to consider. First, our ML approach can be used to create prediction models with high accuracy; however, this comes at the cost of interpretability. The inherent “black box” nature of this approach limits the capability to understand specific coefficients and interaction terms that are used to build the model. Advanced post-hoc analyses may be able to elucidate these specific terms for a more comprehensive understanding of the results. Second, while the diagnosis of PTSD in the NESARC survey is determined via the AUDADIS-IV, the reporting of potentially traumatic events is retrospective and may be unreliable. It is important for future research to incorporate truly prospective methods that do not rely on retrospective self-report of extremely adverse events.

Finally, as mentioned previously, there is an abundance of important information that can be acquired from biological, genetic, physiological, and neurocognitive data that was unavailable in this study. Using a similar ML approach in future studies incorporating these data would shed further light on the high-dimensional interactions between an individual’s environment, genetic makeup, psychological tendencies, physiological processes and brain structure, giving a comprehensive picture of areas to target for prevention and treatment.

## Conclusions

This study used a machine learning approach to identify clinical predictors of and predict new-onset PTSD in a nationally representative community sample. The models achieved exceptional performance in predicting new PTSD onset and identified past Borderline Personality Disorder diagnosis and past depression as two of the most significant predictors in this model. Community-level variables were not significant in our models. This finding may have implications for risk assessment and resource allocation following potentially traumatic events wherein more comprehensive risk assessment profiles may elucidate the likelihood of developing PTSD following an extremely adverse event.

## Data Availability

The datasets analyzed during the current study publicly available at https://nda.nih.gov/niaaa.

## Abbreviations

AUDADIS-IV: Alcohol Use Disorder and Associated Disability Interview Schedule, DSM-IV Version
BART: Bayesian additive regression trees
BPD: Borderline personality disorder
CAPS-DX: Clinically Administered PTSD Scale
DSM-IV-TD: Diagnostic and Statistical Manual of Mental Disorders, fourth edition, text revision
FBI: Federal Bureau of Investigation
GAD: Generalized anxiety disorder
GBM: Gradient boosting machine
GDP: Gross domestic product
IQ: Intelligence quotient
MDD: Major depressive disorder
ML: Machine learning
NIAAA: National Institute on Alcohol and Related Conditions
NESARC: National Epidemiological Survey on Alcohol and Related Conditions
PTSD: Post-traumatic stress disorder
